# Effects of Mechanical Over-Loading on the Properties of Soleus Muscle Fibers, with or without Damage, in Wild Type and *Mdx* Mice

**DOI:** 10.1371/journal.pone.0034557

**Published:** 2012-04-16

**Authors:** Masahiro Terada, Fuminori Kawano, Takashi Ohira, Naoya Nakai, Norihiro Nishimoto, Yoshinobu Ohira

**Affiliations:** 1 Graduate School of Frontier Biosciences, Osaka University, Toyonaka City, Osaka, Japan; 2 Graduate School of Medicine, Osaka University, Toyonaka City, Osaka, Japan; 3 Laboratory of Immune Regulation, Wakayama Medical University, Ibaragi City, Osaka, Japan; University of Texas Health Science Center at Houston, United States of America

## Abstract

Effects of mechanical over-loading on the characteristics of regenerating or normal soleus muscle fibers were studied in dystrophin-deficient (*mdx*) and wild type (WT) mice. Damage was also induced in WT mice by injection of cardiotoxin (CTX) into soleus muscle. Over-loading was applied for 14 days to the left soleus muscle in *mdx* and intact and CTX-injected WT mouse muscles by ablation of the distal tendons of plantaris and gastrocnemius muscles. All of the myonuclei in normal muscle of WT mice were distributed at the peripheral region. But, central myonuclei were noted in all fibers of WT mice regenerating from CTX-injection-related injury. Further, many fibers of *mdx* mice possessed central myonuclei and the distribution of such fibers was increased in response to over-loading, suggesting a shift of myonuclei from peripheral to central region. Approximately 1.4% branched fibers were seen in the intact muscle of *mdx* mice, although these fibers were not detected in WT mice. The percentage of these fibers in *mdx*, not in WT, mice was increased by over-loading (∼51.2%). The fiber CSA in normal WT mice was increased by over-loading (p<0.05), but not in *mdx* and CTX-injected WT mice. It was suggested that compensatory hypertrophy is induced in normal muscle fibers of WT mice following functional over-loading. But, it was also indicated that muscle fibers in *mdx* mice are susceptible to mechanical over-loading and fiber splitting and shift of myonuclei from peripheral to central region are induced.

## Introduction

The dystrophin-deficient (*mdx*) mouse is a well-known animal model for Duchenne muscular dystrophy. The muscles of *mdx* mice are characterized by cycles of muscle fiber necrosis followed by regeneration [1]. The lack of dystrophin in *mdx* muscle causes a reduction in force-generating capacity of muscle, and increases its sensitivity to mechanical stress-induced injury following the application of lengthening (eccentric) contraction [2]. Disruption of dystrophin-glycoprotein complex significantly impairs the membrane integrity or stability during muscle contraction and relaxation. Therefore, the enhanced susceptibility to exercise-induced damage of muscle fibers is observed in *mdx* mice [3]. Further, the regenerating fibers can be easily identified, because they contain conspicuous internal (central) myonuclei [4]. It has been also reported that the appearance of the centronucleation of skeletal muscle fibers was prevented by the inhibition of muscle contractions by limb immobilization, suggesting that muscle contractions play a role in the skeletal muscle degeneration of *mdx* mouse muscles [5].

By mechanical over-loading for 10–12 weeks, the weight of muscle increased approximately double [6], [7]. The maximum isometric tension of muscle did not increase within 1 week after over-loading, but this property increased after long periods of treatment [8]. Mechanical over-loading increased the synthesis of protein [1]. It is known that compensatory hypertrophy is associated with the increase in the size of muscle fibers [9].

However, it is still unclear how the functional over-loading affects the characteristics of mouse soleus muscle fibers. The responses of fiber properties to damage and regeneration in *mdx* and wild type (WT) mouse muscles are not fully elucidated, either. Therefore, the current study was performed to test the hypotheses that responses of fiber properties to damage and/or over-loading are different between *mdx* and WT mice and that damaged fibers are fragile in response to the application of mechanical stress, not lengthening (eccentric) contraction [2].

## Materials and Methods

All experimental procedures were conducted in accordance with the *Guide for the Care and Use of Laboratory Animals* of the Japanese Physiological Society. This study was also approved by the Committee on Animal Care and Use at Graduate School of Medicine, Osaka University (No. 22-071). All surgeries were performed under aseptic conditions with the mice under deep anesthesia (sodium pentobarbital, 5 mg/100 g body weight, *i.p.*).

### Animal care, surgeries, and samplings

Five-week-old male C57BL/10-*mdx* Jic (n = 5) and 4 and 5-week-old male C57BL/6J Jcl (WT, n = 5 each) mice were used. All mice were housed in a controlled environment with 12∶12 hr of light∶dark cycle and the temperature and humidity were maintained at ∼23°C and ∼55%, respectively. Two to three mice were housed in a cage with 11×20 cm and 11 cm height. Solid food (CE-2, Nihon CLEA, Tokyo) and water were supplied *ad libitum*.

In 4-week-old WT mice (n = 5), 5 µl of 0.3 mg/ml cardiotoxin (CTX, Sigma, St. Louis, MO, USA) was injected into soleus muscles bilaterally to induce muscle injury. The CTX is known to damage the plasma membrane of myofibers, but leaves the basal lamina, satellite cells, and nerves intact allowing the rapid and reproducible muscle regeneration [10], [11]. After 7 days of recovery from the CTX injection, CTX-injected WT (n = 5) and other 5-week-old WT and *mdx* mice (n = 5 each) were treated with 14 days of functional over-loading. Modified procedures, reported by Kawano *et al.* [12], were used for the treatment with functional over-loading. To apply the mechanical over-loading for the left soleus muscle, the distal tendons of plantaris and gastrocnemius were ablated in intact *mdx* and WT and CTX-injected WT mice (n = 5 each) under anesthesia by *i.p.* injection of sodium pentobarbital (5 mg/100 g body weight). The neural and vascular supplies were kept intact. The contralateral muscles were kept intact and served as the normal or CTX-injected control. The skin was sutured and the animals were allowed normal ambulation in the cage for 14 days. The behavior and daily activity of these mice in the cages were monitored by video filming during light period.

A single injection of 5-bromo-2′-deoxyuridine (BrdU, Sigma-Aldrich, 100 mg/kg body weight) in phosphate-buffered saline (PBS) was performed *i.p.* 2 days prior to the sampling. At the 14th day after surgery, the mice were anesthetized by *i.p.* injection of sodium pentobarbital (5 mg/100 g body weight). The soleus muscles were sampled bilaterally and cleaned of excess fat and connective tissue. For the longitudinal analyses of single fibers, the muscles sampled from *mdx* and WT mice with and/or without CTX-injection were stored in a freezing medium, named as “cellbanker” (Nihon Zenyaku, Tokyo) at −80 °C.

The control and similarly treated C57BL/10-*mdx* Jic and C57BL/6J Jcl (WT) mice (n = 2 each) were also used additionally for analyses of cross-sectional and longitudinal photographic images of muscle fibers. Soleus muscles were sampled bilaterally. The right muscles were stretched equivalent to an *in vivo* length and pinned on a cork. They were frozen in isopentane cooled with liquid nitrogen. And the mid-portion of the frozen muscle was mounted on a cork perpendicularly by using optimum cutting temperature (OCT) compound (Miles, IN, U.S.A), frozen in liquid nitrogen again, and stored at −80 °C for analyses of the location of myonuclei in the cross-sectional samples. The left muscles were stored in cellbanker (Nihon Zenyaku, Tokyo) at −80 °C for three-dimensional analyses of myonuclear distribution.

### Longitudinal analyses

The muscles stored in the cellbanker were thawed instantly at 35°C. The muscles were placed in Dulbecco's modified Eagle's medium (DMEM, Invitrogen) containing 0.06% type I collagenase, 1% antibiotics, and 10% new-born calf serum (NCS) for 3 hr at 35°C to digest the collagens. The collagenase-treated samples were fixed in 4% buffered-formaldehyde for 10 min and rinsed in PBS. Entire single muscle fibers were isolated from tendon-to-tendon using fine needles. The fibers were collected carefully using pipette to avoid any damage. The collected fibers were separated into three tubes (No. 1-3, 15 fibers in each tube) and immersed in DMEM containing 10% NCS.

For immunohistochemistry, tube No. 1 and 2 were used to label M-cadherin and BrdU to measure the number of quiescent and mitotic active satellite cells, as described previously [13]. The single fibers in these tubes were permeabilized with 1% triton X-100 diluted with PBS for 10 min. These fibers were, then, blocked in 10% goat serum diluted with PBS for 15 min at room temperature. The primary polyclonal antibody specific to M-cadherin (Santa Cruz Biolotechnology, Inc.) and monoclonal antibody specific to BrdU (Becton Dickinson) diluted at 1∶50 with PBS containing 0.5% Tween 20 and 0.5% bovine serum albumin (BSA) was applied to the tube No. 1 and 2, respectively. And the tubes were kept at 4°C for at least 12 hr. The primary antibody was detected by secondary antibody reaction, which was performed for 2 hr at 4°C, using goat anti-rabbit immunoglobulin G (IgG) conjugated to fluorescein (Chemicon International, Inc.) and goat anti-mouse IgG conjugated to fluorescein-isothiocyanate (Jackson Immuno Research) diluted at 1∶100 with 0.5% Tween 20 and 0.5% BSA for the tube No. 1 and 2, respectively. The nuclei (myonuclei+satellite cells) of the antibody-reacted fibers in the tube No. 1 and 2 were further double-counterstained with propidium iodide (PI, 10 µg/ml PBS) for 10 min and Hoechst 33342 (Dojindo Laboratories, 1 µg/ml PBS with 50% glycerol). PI staining was performed for measurement of myonuclear size and observation of branched fibers, and Hoechst staining for observation of myonuclear distribution. Fibers in the tube No. 3 were incubated with α-bungarotoxin in PBS (1∶100) for 10 min to stain neuromuscular junction (NMJ). After staining, the single fibers were rinsed with PBS and stored in PBS at 4 °C until analyses. A cover-glass, with “struts” of hardened nail polish on the corners to minimize fiber compression, was further placed over the samples.

For the three-dimensional analyses of myonuclear distribution, the frozen left muscles, stored in the cellbanker, were also thawed and single muscle fibers were isolated as was stated above. Three-dimensional imaging of soleus muscle fibers was performed using a FV-300 confocal microscope system (Olympus, Japan). The myonuclei were stained with PI (10 µg/ml PBS) for 5 min. The stained fibers were mounted on a slide glass with 50% glycerol in PBS. The fiber was scanned by an argon laser (488 nm of peak wave length) and computerized with a filter set for PI. Such scanning was repeated by moving the stage at 0.1 µm step width and the obtained images were saved as a multiple-TIFF file format. The image file was converted into a gray scale and three-dimensional image was constructed by merging all scanned pictures using 3D Viewer (Ratoc System Engineering).

### Cross-sectional analyses

Cross-sections of the frozen right muscles, mounted on a cork, were cut (10-µm thickness) in a cryostat maintained at −20 °C. These sections were used for the analyses of the distribution of myonuclei. Staining of basal membrane was performed using the primary specific antibody against laminin (Sigma-Aldrich, U.S.A). These sections were, then, blocked in 10% BSA diluted with PBS for 20 min at room temperature. The primary monoclonal antibodies specific for laminin, i.e., primary antibody, anti-laminin diluted at 1∶200 with PBS were applied to these sections, respectively. And the sections were kept at 4°C for at least 12 hr. The primary antibody was detected by secondary antibody reaction, which was performed for 2 hr at 4°C, using both goat anti-rabbit IgG conjugated to fluorescein (Chemicon International, Inc.) diluted at 1∶100 with PBS. After staining, these sections were rinsed with PBS. Immediately, the fibers on a slide glass were covered with a solution containing Hoechst 33342 (Dojindo Laboratories, 1 µg/ml PBS with 50% glycerol) to stain nuclei (myonuclei+satellite cells). A cover-glass, with “struts” of hardened nail polish on the corners to minimize fiber compression, was further placed over the samples. The stained images were analyzed by using a FV-300 confocal microscope with calibrated measurement software (Olympus, Japan). The fibers with or without central nuclei were identified by visual examination.

### Confocal microscopy

**Figure 1:**
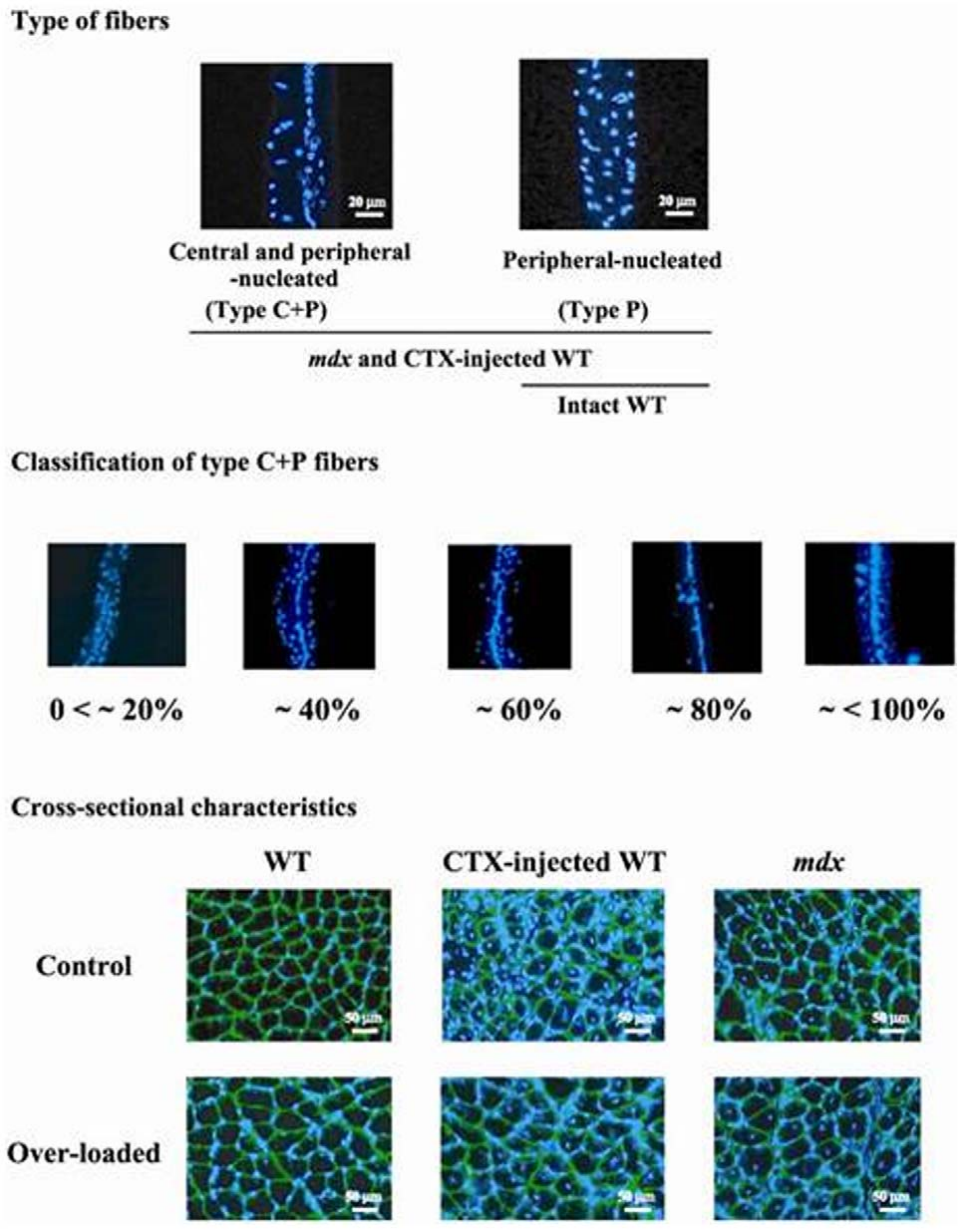
Representative cross-sectional and longitudinal characteristics of soleus muscle fibers in *mdx* and WT mice. Immunohistochemical procedures were performed to stain myonuclei (blue) using Hoechst 33342 and laminin (green) using rabbit anti-laminin antibodies (Sigma, USA). *mdx*: dystrophin-deficient mice, WT: wild type mice, CTX: cardiotoxin, type P and C+P fibers: muscle fibers with myonuclear distribution at only peripheral and both central and peripheral regions, respectively. The characteristics of type C+P fibers were classified into 5 groups according to the percent distribution of central nuclei per single fiber; 0<∼20%, ∼40%, ∼60%, ∼80%, and ∼<100%. Greater differences were also noted in the fiber sizes of *mdx* mouse muscle.

A FV-300 confocal microscope with an argon laser (488 nm of peak wavelength, Olympus) was used to analyze the length and cross-sectional area (CSA) of fibers, number and CSA of the myonucleus, distribution of quiescent and mitotically active satellite cells, and number and location of NMJ. In the Hoechst-stained samples, the fibers with various distribution of myonuclei were classified into two types (Fig. 1) as described previously [14]. Three-dimensional images of PI-stained type P fiber, in which myonuclei were arranged at the peripheral region of fiber only, and type C+P fiber, in which myonuclei were located at both central and peripheral regions, are shown in Figure S1 and S2, respectively. Moreover, number of central and peripheral nuclei was counted and % distribution of central nuclei per single fiber was calculated. Type C+P fibers were classified into 5 groups according to the distribution of central nuclei per single fiber as 0<∼20%, ∼40%, ∼60%, ∼80%, and ∼<100% (Fig. 1). Number of branched fibers was also counted in PI-stained samples. Further, distribution of myonuclei and their number were also analyzed.

The fibers in the tubes No. 1 and 2 were used to measure the fiber CSA and the number and CSA of the myonuclei. First, a maximum-intensity projection rotated orthogonally to the long axis of the fiber was produced from the stack, and the fiber CSA was measured at three non-overlapping regions randomly chosen along the fiber length. The Hoechst-labeled nuclei were counted in the entire single fiber. Myonuclear domain size [15], [16] was also calculated by multiplying the fiber CSA and the fiber length and then dividing by the myonuclear number per fiber. The CSAs of the myonuclei (at least 10 myonuclei in each fiber) was also measured by a given intensity of scanning with PI filter set. In the tube No. 1 and 2, the M-cadherin- and BrdU-labeled satellite cells per fiber were counted to measure the number of quiescent and mitotic active satellite cells, respectively. As for the tube No. 3, the distribution and number of NMJ were measured. The fiber length and the length of 10 consecutive sarcomeres were also measured in each fiber by Normarski optic scan and the fiber CSA and the length of the fiber were normalized at a 2.5-µm sarcomere length. The sarcomere number was calculated as the fiber length divided by a 2.5-µm sarcomere length.

### Statistical analyses

Values are expressed as means ± SEM. Significant differences among groups were determined by using analysis of variance followed by Scheffé's post hoc test. Differences were considered significant at the 0.05 level of confidence.

## Results

### Distribution of central nucleated fibers

**Figure 2:**
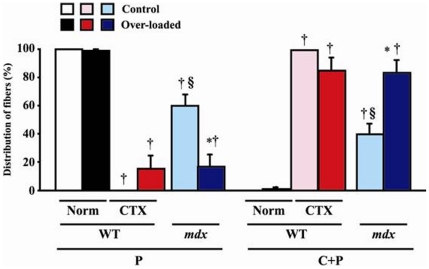
Responses of myonuclear distribution to over-loading and/or CTX injection. The percentages of fibers with different distribution of myonuclei, which were analyzed in whole fiber sampled from tendon-to-tendon, are shown. All of the fibers in the normal muscle without CTX injection (Norm) of WT mice were peripheral-nucleated (type P). Mean ± SEM. *, †, and §: p<0.05 vs. control side, Norm of WT in each type P and C+P fiber, and CTX of WT, respectively. See Figure 1 for other abbreviations.

**Figure 3:**
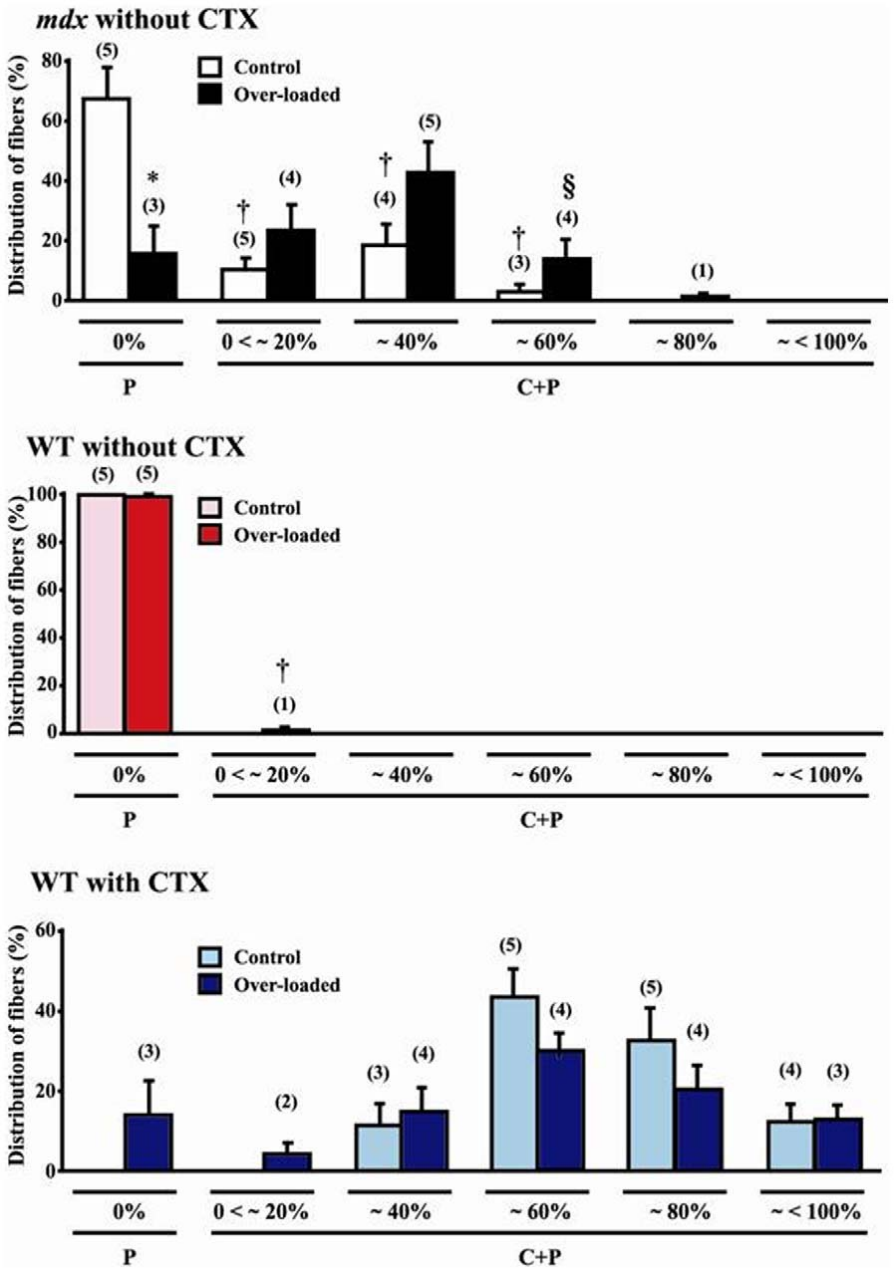
Percent distribution of fibers according to the distribution of central nuclei per single fiber. Mean ± SEM. *, †, and §: p<0.05 vs. control muscle, type P fibers, and ∼40% central-nucleated fibers, respectively. Numbers shown in parentheses indicate the observed animal number out of 5 animals. See Figure 1 for the abbreviations.

Types of fibers were classified into two according to the distribution of myonuclei in peripheral (P) and/or central (C) region of single muscle fiber (Figs. 1, S1, and S2). Muscle fibers with myonuclear distribution at only peripheral and both central and peripheral regions, were described as type P and C+P, respectively. Type C+P fibers were further classified into 5 groups according to the percent distribution of central nuclei per single fiber, as 0<∼20%, ∼40%, ∼60%, ∼80%, and ∼<100%. All of the fibers in the control side of normal WT mice were type P (Figs. 1 and 2). But type C+P fibers (0.9%) were noted in the over-loaded muscle of WT mice (Fig. 2). In response to CTX-injection, all fibers in WT mice changed to type C+P. However, shift from type P to C+P was insignificantly inhibited by mechanical over-loading (14.9%). The distributions of type P and C+P fibers in *mdx* mice were 60.3 and 39.7%, and type P fibers were further shifted to type C+P by over-loading (43.6%, p<0.05). The data shown in Figure 2 and 3 include those from the branched fibers.

Following the functional over-loading, the distribution of type P fibers decreased (77%, p<0.05) and that of type C+P fibers with 0<∼20%, ∼40%, and ∼60% central nuclei insignificantly increased in *mdx* mice (Fig. 3). And the distribution of fibers with ∼60% central nuclei was less than that of fibers with ∼40% central nuclei after over-loading (p<0.05). The distribution of type C+P fibers with 0<∼20%, ∼40%, and ∼60% central nuclei without application of over-loading was significantly less than that of type P fibers (p<0.05). And fibers with ∼80% central nuclei were seen after over-loading in 1 out of 5 *mdx* mice. In CTX-injected WT mice, the distribution of type C+P fibers was in the order of ∼60%>∼80%>40% = ∼<100%>0<∼20% central nuclei. And the percentage of fibers with ∼60% and ∼80% central nuclei tended to decrease after functional over-loading insignificantly. The distribution of fibers with 0<∼20% central nuclei was seen in 2 out of 5 CTX-injected mice following over-loading. No clear differences in the behavior and activity levels in the cages were noted between *mdx* and WT mice with and without muscle damage (data not shown).

### Fiber size

**Figure 4:**
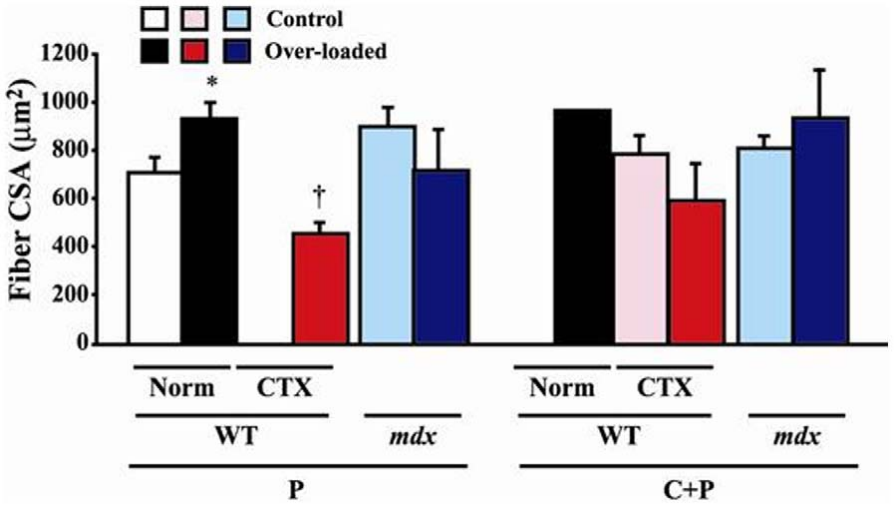
Fiber cross-sectional area (CSA) in WT, *mdx*, and CTX-injected WT mice. Mean ± SEM. * and †: p<0.05 vs. control muscle and Norm of WT, respectively. See Figure 1 and 2 for other abbreviations.

The fiber CSA in WT mice increased by 31.8% after over-loading (p<0.05, Fig. 4). However, the CSA of type P fibers in *mdx* mice was insignificantly decreased by 21.3% following functional over-loading, although that of type C+P fibers was insignificantly increased by 15.1%. The mean CSA of CTX-injected plus over-loaded type P fibers in WT mice was less than that of over-loaded normal muscles (p<0.05). The length of type C+P fibers of *mdx* mice without over-loading was shorter than those of CTX-injected WT mice (p<0.05, Fig. S3). However, the mean fiber length was identical between most of the groups.

### Characteristics of myonuclei

**Figure 5:**
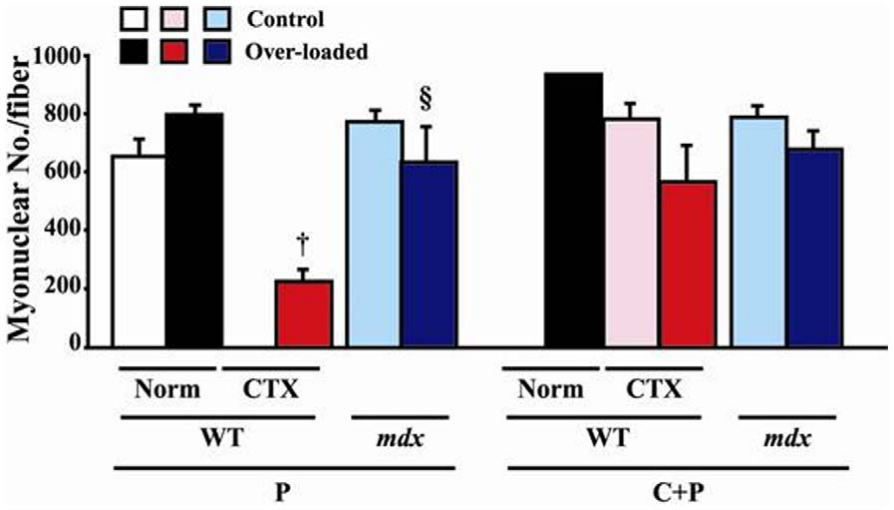
Myonuclear number per fiber in WT, *mdx*, and CTX-injected WT mice. Mean ± SEM. † and §: p<0.05 vs. Norm of WT and CTX of WT, respectively. See Figure 1 and 2 for the abbreviations.

The myonuclear number per single fiber of both WT and *mdx* mice did not change significantly in response to over-loading (Fig. 5). The whole myonuclear number in over-loaded type P fibers of CTX-injected WT mice was significantly less than the over-loaded fibers without CTX injection (p<0.05). A significantly greater number of myonuclei was noted in the over-loaded muscle of *mdx* mice than that of CTX-injected plus over-loaded muscle of WT mice (p<0.05). The response of the myonuclear number in *mdx* mice was identical between type P and C+P fibers. The number of myonuclei was also counted in the branched fibers.

Statistically significant differences were not observed in the myonuclear domain size between any groups, although insignificantly greater value was noted in the over-loaded type P fibers of CTX-injected WT mice (Fig. S4). This phenomenon may be caused by the greater loss of myonuclei by combination of CTX injection and over-loading (Fig. 5), even though the fiber CSA was also smaller (Fig. 4). Myonuclear CSA in all groups tended to be increased by functional over-loading, but not significantly (Fig. S5). These tendencies were similar between type P and C+P fibers. The differences in the responses between two species were not observed, either.

### Distribution of branched muscle fibers

**Figure 6:**
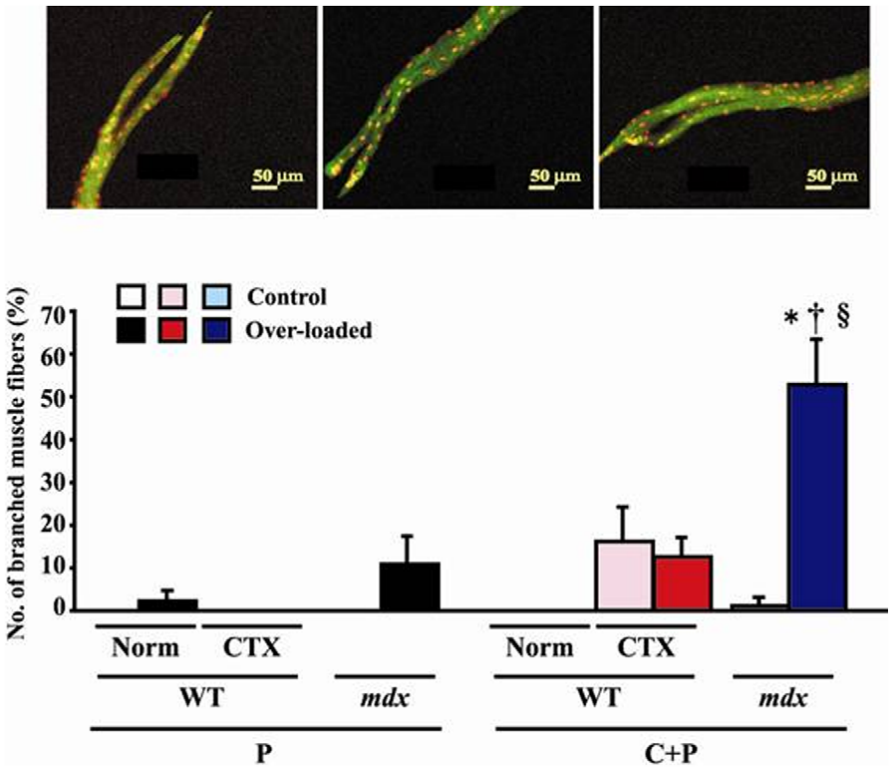
Photographs and percent distribution of branched fibers. Red and yellow dots show the myonuclei and green color shows the cytoplasm. Mean ± SEM. *, †, and §: p<0.05 vs. control muscle, Norm of WT, and CTX of WT. See Figure 1 and 2 for the abbreviations.

In general, no branched fibers were noted in the normal control and/or CTX-injected muscle of WT and *mdx* mice without application of mechanical load, although branched fibers were seen in type C+P fibers of *mdx* (1.4%) and CTX-injected WT mice (18.6%), respectively (Fig. 6). The over-loading-related increases in type P fibers of normal WT mouse muscle without CTX injection (2.5%) and *mdx* mice (10.5%) were not significant and the percentage of type C+P fibers of CTX-injected WT mice was stable. But approximately 51.2% increase of type C+P branched fibers was noted in the over-loaded *mdx* muscle (p<0.05).

### Characteristics of satellite cells

The number of mitotically active satellite cells was not influenced significantly by over-loading (Fig. S6). These cells in type P fibers of CTX-injected WT and *mdx* mice, as well as the normal type C+P fibers of WT mice, disappeared in response to increased mechanical loading, even though those fibers were still existed (Fig. 2). Higher distribution of these cells was noted in both type P and C+P fibers of the control *mdx* mouse muscle (p>0.05). Quiescent satellite cells in type P fibers of CTX-injected WT and *mdx* mice, as well as the normal type C+P fibers of WT mice, were noted after over-loading (Fig. S6), even though mitotic active satellite cells were not observed in these fibers, as was stated above. The numbers of these cells in both type P and C+P fibers of WT and *mdx* mice were not significantly influenced by CTX injection and over-loading.

### Distribution of NMJ

All of the fibers in whole groups had a single NMJ at the central region (data not shown).

## Discussion

It is thought that muscle fibers with central nuclei are at the emergence or regeneration stage generally [4]. Previous study reported that the immature myogenic cells, such as myoblasts and myotubes, were centrally nucleated [17]. The CSA and length of fibers, total myonuclear number, and domain size were identical among the type C+P and P fibers in the present study, suggesting that the fibers with central nuclei were morphologically similar to the mature fibers. The responses of soleus muscle fibers of *mdx* and intact and CTX-injected WT mice to increase of mechanical loading were also studied. One of the hypotheses that responses of fiber properties to damage and/or over-loading are different between *mdx* and WT mice was accepted. Further, prominent fiber splitting in response to mechanical loading was noted in the muscle of *mdx* mice. But the muscle fibers of WT mice, even with damage, were resistive. Since the behavior and activity levels in the cage were similar between two kinds of mice with and without damage, such responses of fiber properties could be induced by functional over-loading, not daily activity level.

### Distribution of fibers

Shift of localization of myonuclei from peripheral to central region was induced in *mdx* mice following functional over-loading. In CTX-injected WT mice, the myonuclei also tended to move from central to peripheral region in response to over-loading. These results indicated that muscle fiber degeneration in *mdx* mice was activated by over-loading. Such phenomena were also supported by the increased number of branched fibers. On the contrary, the functional over-loading tended to activate the recovery from the damage in CTX-injected WT mice. Approximately 14.9% of fibers shifted from type C+P to P (p>0.05).

### Fiber size

Hypertrophy of fibers was induced in response to mechanical over-loading in intact (normal) WT mouse muscles, but not in *mdx* and CTX-injected mice. Compensatory over-loading by ablation of synergists is known to result in increased muscle mass [18], [19], [20], [21], [22], [23]. It was reported that muscle became considerably larger at an early stage (7th day) of over-loading, but the mean diameters of the fiber did not change [24]. In addition, Richard-Bulteau *et al.* [25] observed numerous regenerating fibers with small diameter in soleus muscle of rat 21 days after notexin injection. They also reported that these regenerating fibers were enlarged after the forced running exercise using a treadmill, but the size of these enlarged regenerating fibers was still smaller than that of non-injured fibers. These results may suggest that injured or regenerating fibers may be less sensitive to functional over-loading, relative to normal fibers. Prominent response of fiber CSA to over-loading was not observed in *mdx* mice, although the CSA in type P fibers of normal WT mice increased, as was stated above. Such phenomena in the muscle of *mdx* mice may be related to the fiber splitting seen following over-loading.

### Characteristics of myonuclei

None of the characteristics of myonuclei, such as number, size, and domain, changed significantly by functional over-loading, although there was some insignificant trend in some parameters. Previously, we reported that these characteristics were identical regardless of the distribution of myonuclei in soleus muscle fiber of *mdx* mice [26]. It was reported that the number of myonuclei was decreased and myonuclear size was increased in association with fiber atrophy caused by inhibited utilization of muscle in adult mice and rats [13], [27]. However, it was also reported that 14-day over-loading of soleus muscle did not affect the myonuclear number in male Wistar rats [12]. The myonuclear number in the over-loaded WT mouse muscle did not increase significantly in the present study, either, although MaCarthy et al. [28] reported that the number of myonuclei was increased after 2 weeks of over-loading due to fusing of satellite cells. The results in the present study may suggest that the hypertrophy of muscle fibers seen in the normal muscle of WT mice in response to over-loading may be related to the increased number of myonuclei (+25%, p>0.05), not necessarily the improved function of each myonucleus, since the myonuclear domain size was stable.

### Distribution of branched muscle fibers

The percent distribution of branched fibers was significantly increased in type C+P fibers of *mdx* mice in response to functional over-loading. It is known that branched or splitting muscle fibers could be found after muscle damage [29], [30], [31], [32], [33]. In particular, Vaughan *et al.* [24] reported that splitting fibers were not found after 7 days of over-loading, but fibers with splitting distally into two or more smaller fibers were frequently noted after 55 and 208 days. It was also reported that sarcomeres were disorganized in damaged muscle fibers [34]. In some splitting fibers, sarcomere patterns were disrupted and Z-discs were damaged [24].

Increased splitting of fibers following mechanical over-loading, not by application of lengthening (eccentric) contraction [2], was seen only in *mdx* mice. Exercise-induced damage [3] and, on the contrary, immobilization-related inhibition of fiber damage [5] are also reported elsewhere. The results from the current study and published papers [2], [3], [5] clearly suggested that muscle fibers in *mdx* mice are fragile. It is interesting that such load-dependent splitting was not noted in the injured muscle of WT mice. The branched portion of these fibers had both peripheral and central nuclei. And the localization of NMJ in these fibers was similar to those of not-branched fibers. The data suggested that approximately 43.6% of type P fibers in *mdx* mice were shifted to type C+P fibers in response to over-loading. It is suggested that load-dependent damage or splitting may be induced mainly in type P fibers and the distribution of myonuclei may be shifted to central region of fiber accordingly.

### Muscle satellite cells

It has been clearly shown that satellite cells play crucial roles in muscle regeneration [13], [35], [36], [37], [38]. Schefer *et al.* [27] reported that there was no difference in the number of satellite cells in adult *mdx* mice and controls. However, it was also reported that satellite cells from *mdx* mice were more sensitive to fibroblast growth factor in culture than those from normal mice [39]. Further, the differentiation of satellite cells obtained from *mdx* mice was accelerated by the higher expression of myocyte enhancer factor 2 (MEF2) [40]. It was speculated that satellite cells in *mdx* mouse muscles may be always more active than those in WT mice. Therefore, we also counted the numbers of both mitotically active and quiescent satellite cells of the muscle fibers in the present study. However, no significant responses in the number of satellite cells were noted in response to over-loading, although the mean number of mitotically active cells in non-injured intact type P muscle fibers of *mdx* mice were greater than that of WT mice (p>0.05, Fig. S6). Injection of 5-ethynyl-2′-deoxyuridine (EdU, 0.1 mg/30 g body weight) on days 2, 3, and 4 and administration of BrdU (0.8 mg/ml) in drinking water for 8 consecutive days after injury were performed to label the satellite cells by Lepper et al. [35]. McCarthy et al. [28] also administered BrdU in drinking water for 2 weeks. Therefore, it may be possible that continuous administration of BrdU may cause the increase of Pax7-positive satellite cells. On the contrary, a single *i.p.* injection of BrdU has been performed in our group [13], [41], [42] 2 days prior to the sampling. Such methodological differences may be one of the causes for such discrepancy. The roles of satellite cells in the plasticity of muscle fibers of *mdx* mice are still unclear.

### Conclusion and Perspective

Effects of 14-day mechanical over-loading on the characteristics of regenerating or normal soleus muscle fibers were studied in *mdx* and WT mice. Although all myonuclei in normal muscle fibers of WT mice were distributed at the peripheral region only, central myonuclei were noted in all fibers during recovery from CTX injection. Many fibers of *mdx* mice had central myonuclei and the distribution of such fibers was increased following mechanical over-loading, suggesting a shift of myonuclei from peripheral to central region. The percentage of branched fibers, with both central and peripheral myonuclei, was increased by 51.2% following over-loading in *mdx*, but not in WT, mice, suggesting that muscle fibers of *mdx* mice are susceptible to elevation of mechanical stress. Compensatory hypertrophy was induced in only normal WT mouse fibers by over-loading. The NMJ was seen at the central region of fiber, even in the regenerating fibers of CTX-injected WT and *mdx* mice. Further, the length, total sarcomere number, and activation of satellite cells in single muscle fibers, sampled from tendon-to-tendon, were identical between all groups. Therefore, these results might suggest that the basal lamina, satellite cells, and nerve terminal remained intact even after CTX injection, as was reported elsewhere [10], [11].

## Supporting Information

Figure S1 **Three-dimensional structure of muscle fiber with myonuclei at peripheral region only.**
(MPG)

Figure S2 **Three-dimensional structure of muscle fiber with myonuclei at both peripheral and central region.**
(MPG)

Figure S3 **Length and sarcomere number in soleus muscle fibers.** Mean ± SEM. §: p<0.05 vs. CTX of WT in type C+P fibers. WT: wild type mice, *mdx*: dystrophin-deficient mice, CTX: cardiotoxin-injected, Norm: normal (without CTX injection), P and C+P: muscle fibers with myonuclear distribution at peripheral region only and both central and peripheral regions, respectively.
(TIF)

Figure S4 **Myonuclear domain in WT,** ***mdx*** **, and CTX-injected WT mice.** Mean ± SEM. See Figure S3 for the abbreviations.
(TIF)

Figure S5 **Myonuclear cross-sectional area (CSA) in WT,** ***mdx*** **, and CTX-injected WT mice.** Mean ± SEM. See Figure S3 for other abbreviations.
(TIF)

Figure S6 **Number of satellite cells (SCs) in WT,** ***mdx*** **, and CTX-injected WT mice.** Mitotically active satellite cells were not observed in the over-loaded type P muscle fibers in WT mice following CTX injection. Further, these cells were not seen in the over-loaded type P fibers of *mdx* mice and type C+P fibers of WT mice, either. Mean ± SEM. See Figure S3 for other abbreviations.
(TIF)
