# Supplementary figures and images for: Effects of Mechanical Over-Loading on the Properties of Soleus Muscle Fibers, with or without Damage, in Wild Type and Mdx Mice
Source: PLoS One. 2012 Apr 16;7(4):e34557. doi: 10.1371/journal.pone.0034557 (PMC3327707; doi:10.1371/journal.pone.0034557)

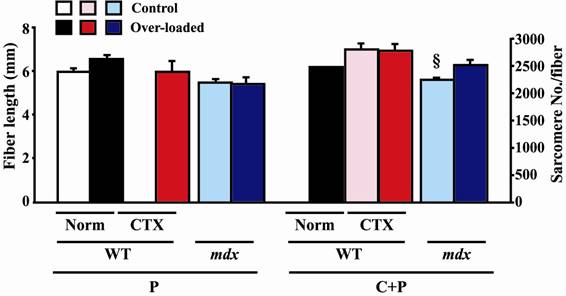

Supplement: Figure S3 — Length and sarcomere number in soleus muscle fibers. Mean ± SEM. §: p<0.05 vs. CTX of WT in type C+P fibers. WT: wild type mice, mdx: dystrophin-deficient mice, CTX: cardiotoxin-injected, Norm: normal (without CTX injection), P and C+P: muscle fibers with myonuclear distribution at peripheral region only and both central and peripheral regions, respectively. (TIF) [file pone.0034557.s003.tif]

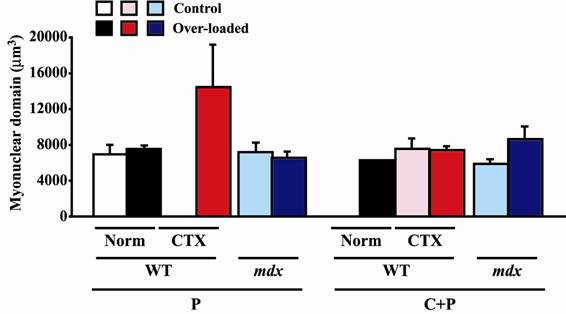

Supplement: Figure S4 — Myonuclear domain in WT, mdx , and CTX-injected WT mice. Mean ± SEM. See Figure S3 for the abbreviations. (TIF) [file pone.0034557.s004.tif]

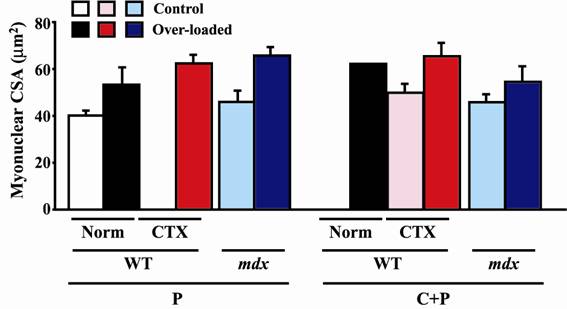

Supplement: Figure S5 — Myonuclear cross-sectional area (CSA) in WT, mdx , and CTX-injected WT mice. Mean ± SEM. See Figure S3 for other abbreviations. (TIF) [file pone.0034557.s005.tif]

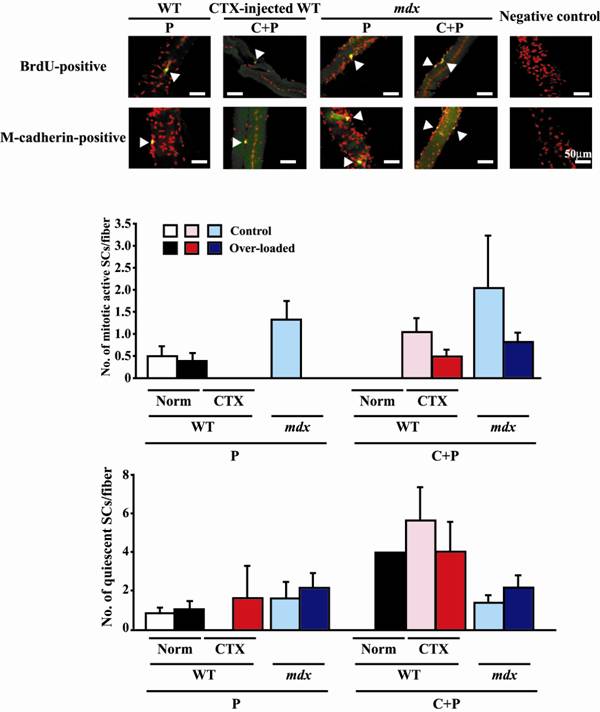

Supplement: Figure S6 — Number of satellite cells (SCs) in WT, mdx , and CTX-injected WT mice. Mitotically active satellite cells were not observed in the over-loaded type P muscle fibers in WT mice following CTX injection. Further, these cells were not seen in the over-loaded type P fibers of mdx mice and type C+P fibers of WT mice, either. Mean ± SEM. See Figure S3 for other abbreviations. (TIF) [file pone.0034557.s006.tif]
